# Modulation of the cardiac sodium/bicarbonate cotransporter by the renin angiotensin aldosterone system: pathophysiological consequences

**DOI:** 10.3389/fphys.2013.00411

**Published:** 2014-01-17

**Authors:** Verónica C. De Giusti, María C. Ciancio, Alejandro Orlowski, Ernesto A. Aiello

**Affiliations:** Facultad de Ciencias Médicas, Centro de Investigaciones Cardiovasculares, Universidad Nacional de La Plata, CONICET-La PlataLa Plata, Argentina

**Keywords:** sodium bicarbonate cotransporter, angiotensin II, aldosterone, hypertrophy, heart

## Abstract

The sodium/bicarbonate cotransporter (NBC) is one of the major alkalinizing mechanisms in the cardiomyocytes. It has been demonstrated the existence of at least two functional isoforms, one that promotes the co-influx of 1 molecule of Na^+^ per 1 molecule of HCO^−^_3_ (electroneutral isoform; NBCn1) and the other one that generates the co-influx of 1 molecule of Na^+^ per 2 molecules of HCO^−^_3_ (electrogenic isoform; NBCe1). Both isoforms are important to maintain intracellular pH (pH_*i*_) and sodium concentration ([Na^+^]_*i*_). In addition, NBCe1 generates an anionic repolarizing current that modulates the action potential duration (APD). The renin-angiotensin-aldosterone system (RAAS) is implicated in the modulation of almost all physiological cardiac functions and is also involved in the development and progression of cardiac diseases. It was reported that angiotensin II (Ang II) exhibits an opposite effect on NBC isoforms: it activates NBCn1 and inhibits NBCe1. The activation of NBCn1 leads to an increase in pH_*i*_ and [Na^+^]_*i*_, which indirectly, due to the stimulation of reverse mode of the Na^+^/Ca^2+^ exchanger (NCX), conduces to an increase in the intracellular Ca^2+^ concentration. On the other hand, the inhibition of NBCe1 generates an APD prolongation, potentially representing a risk of arrhythmias. In the last years, the potentially altered NBC function in pathological scenarios, as cardiac hypertrophy and ischemia-reperfusion, has raised increasing interest among investigators. This review attempts to draw the attention on the relevant regulation of NBC activity by RAAS, since it modulates pH_*i*_ and [Na^+^]_*i*_, which are involved in the development of cardiac hypertrophy, the damage produced by ischemia-reperfusion and the generation of arrhythmic events, suggesting a potential role of NBC in cardiac diseases.

## Introduction

The adequate regulation of intracellular pH (pH_*i*_) is essential for the heart. Fall of pH_*i*_ usually occurs in cardiac myocytes, as during changes in heart rate (Bountra et al., 1988; Elliott et al., 1994), but also a major reduction occurs during pathological conditions, such as myocardial ischemia (Steenbergen et al., 1977; Garlick et al., 1979). Two sarcolemmal alkalinizing ion transporters mediate the acid-extrusion in order to maintain pH_*i*_ near to 7.2, either exporting H^+^ (Na^+^/H^+^ antiporter; NHE-1), or introducing HCO^−^_3_ into the cell, (Na^+^/HCO^−^_3_ cotransporter; NBC).

It has been described at least two functional isoforms of NBC in the heart: the electroneutral NBC, NBCn1, which promotes the co-influx of 1 molecule of Na^+^ per 1 molecule of HCO^−^_3_, and the electrogenic NBC, NBCe1, which introduces 1 molecule of Na^+^ per 2 molecules of HCO^−^_3_.

It has been demonstrated that the increase in the intracellular sodium concentration ([Na^+^]_*i*_) generated by these alkalinizing transporters promotes the Na^+^/Ca^2+^ exchanger to work in its reverse mode (NCXr), leading to an increase in the intracellular calcium concentration ([Ca^2+^]_*i*_), which is involved in the pathogenesis of several cardiac diseases as hypertrophy, the damage produced by ischemia-reperfusion and the generation of arrhythmia.

The renin-angiotensin-aldosterone system (RAAS) represents one of the main endocrine systems that regulate cardiac pathophysiology. Moreover, it is well-recognized that both angiotensin II (Ang II) and aldosterone are expressed locally in the heart, exerting their effects in a paracrine and/or autocrine manner. In this regard, it has been described that Ang II and aldosterone directly stimulate the cardiac NHE-1, which is involved in the positive inotropic response (Perez et al., 2003; Caldiz et al., 2011) and cardiac hypertrophy (Cingolani et al., 2008, 2013) produced by these hormones. In addition, in the last years it has been demonstrated that NBC activity is regulated by Ang II (Baetz et al., 2002; De Giusti et al., 2009, 2010).

In the present review the evidence about the regulation of NBC by RAAS and the potential importance of NBC in the development and maintenance of cardiac diseases mediated by RAAS will be discussed.

## Role of the cardiac NBC in pH_*i*_ and [Na^+^]_*i*_ regulation

At present it is known that NBC is responsible for 40–50% of total acid extrusion in cardiac myocytes under physiological conditions at pH_*i*_ near resting values, when total acid extrusion is low (Lagadic-Gossmann et al., 1992; Camilion De Hurtado et al., 1995). However, it is important to recognize that, although both transporters are equally operative at pH_*i*_ close to basal (Le Prigent et al., 1997; Vaughan-Jones et al., 2006, 2009; De Giusti et al., 2009), at acidic pH_*i*_ (near to 6.8) the relative importance of NBC is only of 30% against the 70% of NHE-1 (Baetz et al., 2002; Vaughan-Jones et al., 2006; De Giusti et al., 2009).

**Figure 1:**
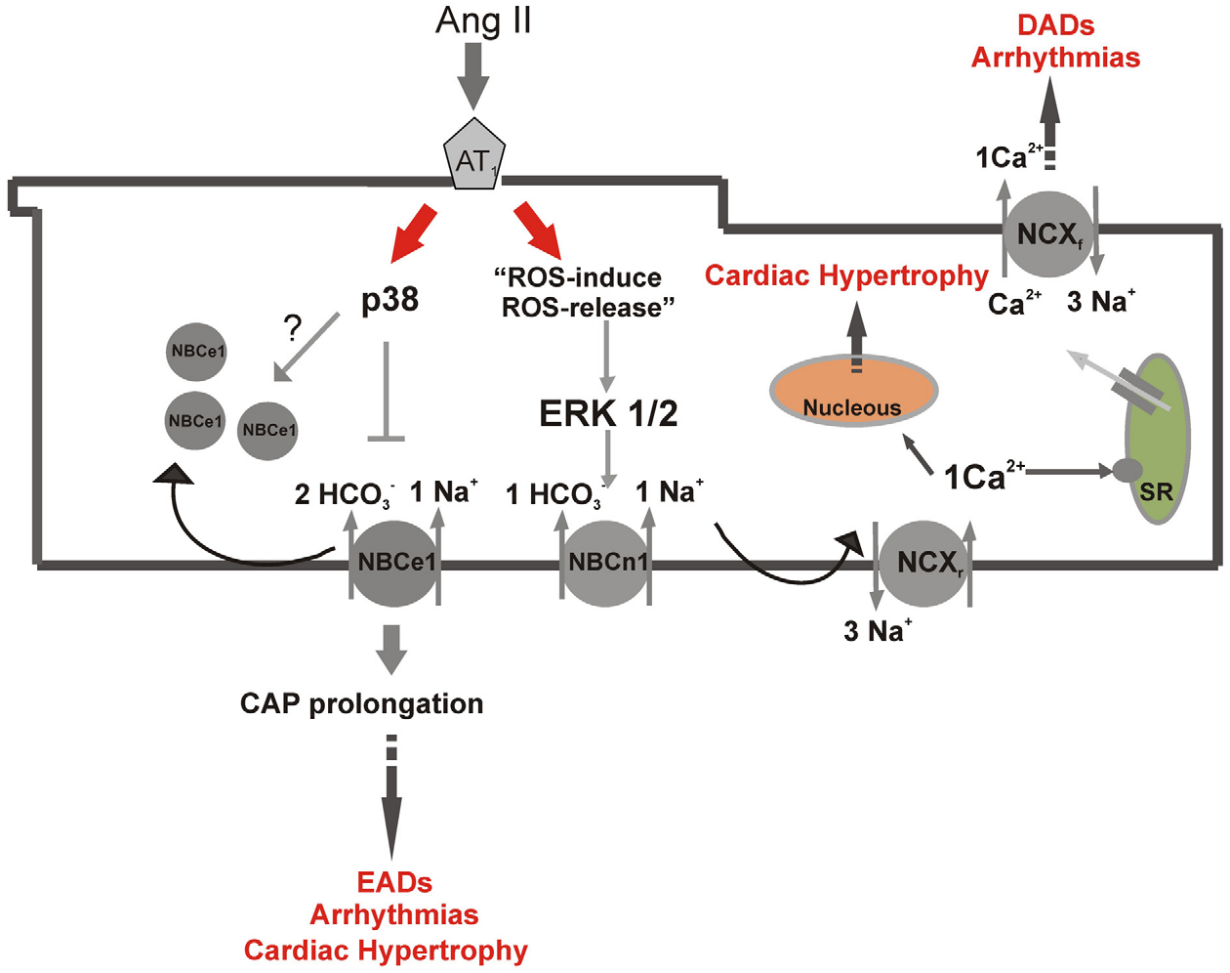
**Differential NBC isoforms regulation by angiotensin II**. Scheme of parallel Ang II-pathways in a ventricular myocyte, showing NBCn1 stimulation and NBCe1 inhibition and the possible implications of these regulations in the development of cardiac pathologies, as hypertrophy and arrhythmias. p38, p38 kinase; ERK ½, ERK kinase; EADs, early after depolarizations; DADs, delay after depolarizations; SR, sarcoplasmatic reticulum; CAP, cardiac action potential; NCXr, reverse mode of sodium/calcium exchanger; NCXf, forward mode of sodium/calcium exchanger.

As it has been established for the NHE-1, it has been demonstrated that NBC increases [Na^+^]_*i*_ (Yamamoto et al., 2005; Vaughan-Jones et al., 2006). The increase in [Na^+^]_*i*_ is crucial for cardiac pathophysiology because, as it is well-known, it stimulates NCXr, leading to an increase in [Ca^2+^]_*i*_ (Rothstein et al., 2002; Bril, 2003; Perez et al., 2003; Aiello et al., 2005), process which is involved in Ang II-induced positive inotropic effects (Aiello et al., 2005) and cardiac hypertrophy (Dulce et al., 2006; Cingolani et al., 2008). In this regard, it was proposed that this phenomenon might be involved in NBC-induced cardiac diseases (Khandoudi et al., 2001; Bril, 2003; Baartscheer and Van Borren, 2008; De Giusti et al., 2010) (Figure 1).

In addition, since it was suggested that the mineralocorticoid receptors (MR) appears to be located downstream of Ang II in the chain of intracellular signals leading to the slow force response (SFR) (Caldiz et al., 2011), the activation of this receptors by aldosterone could also be implicated in the regulation of cardiac contractility. Consistently, an aldosterone-induced positive inotropic effect has been previously reported in rat myocardium (Barbato et al., 2002, 2004).

**Figure 2:**
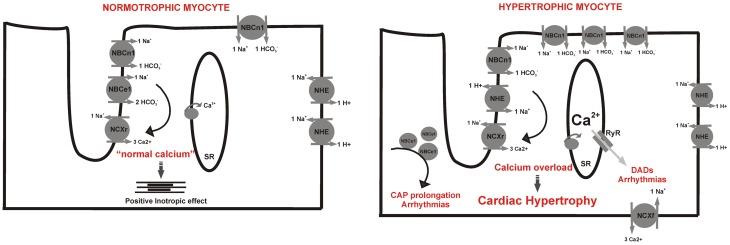
**Potential redistribution of membrane transporters during cardiac hypertrophy. Left**, schematic distribution in the normotrophic cardiac myocyte: both NBC isoforms are mainly expressed in t-tubules in relationship with NCX maintaining a “normal calcium” space near the SR whereas NHE-1 is expressed in intercalated disks. **Right**, hypertrophic myocyte where the NHE-1 and NBCn1 are overexpressed and homogeneously distributed along sarcolemmal membrane, and the trafficking of NBCe1 to the membrane is disturbed leading to its reduced activity. In this scenario there is cytosolic and SR calcium overload which leads to hypertrophy and cardiac arrhythmias. EADs, early after depolarizations; DADs, delay after depolarizations; SR, sarcoplasmatic reticulum; CAP, cardiac action potential; NCXr, reverse mode of sodium/calcium antiporter.

Recently, it was demonstrated that NBCe1 is homogeneously physically and functionally localized in lateral sarcolemma, intercalated disc and especially in the transverse tubules, co-localized with the NCX (Garciarena et al., 2013a). In contrast, NHE-1 is expressed and functionally active only at intercalated discs and lateral surface membrane (Garciarena et al., 2013a). Taking into account the stoichiometry of NBCe1, claimed as a “Na^+^- sparing” bicarbonate transporter, it is feasible to anticipate that this selective distribution of the alkalinizing transporters and their relationship with the NCX may help to reduce the possibility of local Ca^2+^ overload near the sarcoplasmatic reticulum (SR), as recently suggested by Dr. Vaughan-Jones's group (Garciarena et al., 2013a). Interestingly, as it will be further discussed in this review, our group recently showed that NBCe1 activity is impaired whereas NBCn1 is overexpressed in the hypertrophied myocytes of spontaneous hypertensive rats (SHR) (Orlowski et al., 2013). It is also known that NHE-1 activity is increased in SHR rats (Perez et al., 1995). More importantly, in a model of cardiac hypertrophy due to NHE-1 overexpression, the NHE-1 is distributed all around the sarcolemma (Nakamura et al., 2008), further suggesting that the pathological remodeling of these transporters could be, at least in part, responsible for the [Na^+^]_*i*_ and [Ca^2+^]_*i*_ overload-mediated cardiac hypertrophy (Garciarena et al., 2013b) (Figure 2).

## Different participation of NBCn1 and NBCe1 in pH_*i*_ regulation

Cardiac NBC was initially described by Lagadic-Gossmann et al. as an electroneutral transporter (at present known as NBCn1) (Lagadic-Gossmann et al., 1992). Some years later Dr. Cingolani's group demonstrated that cardiac NBC also exhibits an electrogenic behavior (at present called NBCe1) (Camilion De Hurtado et al., 1995). Finally, it was demonstrated that both isoforms coexist in the heart and the fact that the NBC can mediate either the electroneutral or electrogenic bicarbonate transport enlarged the spectrum of research around it.

It has been described and characterized the rat and cat cardiac NBCe1 current as an anionic bicarbonate and sodium-dependent current which reversed at around −85 mV (I_NBC_) (Aiello et al., 1998; Villa-Abrille et al., 2007). Reversal potential for electrogenic NBC was also measured and estimated for rabbit, mouse and guinea pig, with values ranging from −98 to −106 mV (Yamamoto et al., 2005; Vaughan-Jones et al., 2006). It has also been described the influence of NBCe1 in the configuration of the cardiac action potential (CAP) (Aiello et al., 1998; Villa-Abrille et al., 2007). Using the patch-clamp technique, it was demonstrated that the change of the extracellular solution from a HEPES- (HCO^−^_3_-free solution) to a HCO^−^_3_-containing solution, hyperpolarized resting membrane potential (RMP) by 3–5 mV and evoked a 25% CAP shortening, both in rat (Aiello et al., 1998) and cat (Villa-Abrille et al., 2007) ventricular myocytes. Furthermore, Ang II inhibits NBCe1 current and prolongs CAP (De Giusti et al., 2010). In this regard, it is well-known that CAP prolongation is associated either as the initiator (Lebeche et al., 2006) or the consequence of the cardiac hypertrophy and arrhythmogenesis (Fischer et al., 2007; Weisser-Thomas et al., 2007). The outward current mediated by NBCe1, which under normal conditions shortens the CAP, might represent a protective aspect for the myocyte.

Important for the improvement of the knowledge about NBC, it was first described a selective blocker of the transporter (Ch'en et al., 2008), and some years later, specific antibodies against the NBCe1 that allowed the discrimination of the participation of each isoform in pH_*i*_ regulation were generated in our laboratory (De Giusti et al., 2011b). Two different and selective functional antibodies against NBCe1 were produced and characterized that were called a-L3 and a-L4 because they recognized the extracellular loop 3 and loop 4, respectively. Interestingly, these antibodies exhibited opposite effects on NBCe1 function, a-L3 totally inhibits NBCe1 activity, while a-L4 has an excitatory effect on it. In such research it was confirmed that NBCe1 is the only functional electrogenic alkalinizing mechanism in normal cardiac ventricular myocytes. Moreover, since a-L3 reduced proton efflux (*J*_*H*_) during the recovery from intracellular acidosis in an extracellular medium in the presence of bicarbonate and cariporide (NHE-1 blocker) by approximately 50%, it could be speculated that, at least in rat and cat cardiomyoytes, both isoforms of the NBC, NBCe1, and NBCn1, exhibit an equivalent contribution to the regulation of pH_*i*_ under physiological conditions (De Giusti et al., 2011b; Orlowski et al., 2013).

## Renin angiotensin aldosterone system: endocrine vs. paracrine system

Ang II is an octapeptide that classically was known to be synthesized from Ang I by the angiotensin-converting enzyme (ACE) present in the endothelial vessels in response to high levels of aldosterone, forming the endocrine system known as renin-angiotensin-aldosterone-system (RAAS). However, at present it is well-recognized that Ang II is produced and secreted locally in the heart (Husain et al., 1994; Shyu et al., 2001). Furthermore, it was shown that >75% of cardiac Ang II was synthesized locally, and that its source was also *in situ*-synthesized Ang I (De Mello and Danser, 2000). Although still controversial (Silvestre et al., 1998, 1999; Takeda et al., 2000; Gomez-Sanchez et al., 2004; Chai and Danser, 2006), it has been also demonstrated that aldosterone synthase exists in the myocytes (Silvestre et al., 1998), supporting the existence of a local RAAS (Varagic and Frohlich, 2002). Dr. Sadoshima's group has shown that Ang II generates cardiac hypertrophy in response to myocardial stretch and secretion from intracellular vesicles (Sadoshima et al., 1993; Sadoshima and Izumo, 1996). Furthermore, Dr. Cingolani's group has deeply investigated the presence of this autocrine pathway as responsible for the SFR to myocardial stretch, proposing the NHE-1 as the final effector. (Cingolani et al., 2001, 2003). Moreover, aldosterone has been shown to activate NHE-1, (De Giusti et al., 2011a) and to increase NHE-1 expression, inducing left ventricular hypertrophy (Karmazyn et al., 2003), independently from its classical effects on regulation of renal Na^+^ excretion and blood pressure (Qin et al., 2003; Yoshida et al., 2005; Diez, 2008). In addition, the investigation of the role of aldosterone in cardiac pathophysiology has gained increasing interest in the last few years due to relevant results obtained from clinical studies, particularly RALES (Randomized Aldactone Evaluation Study), EPHESUS (Eplerenone Post–acute Myocardial Infarction Heart Failure Efficacy and Survival Study), and EMPHASIS-HF, in which antagonists of the MR importantly reduced mortality in patients with left ventricular dysfunction independently of the values of blood pressure (Pitt et al., 1999, 2001; London et al., 2003).

Classically, aldosterone enters the cells and binds to the MR located mainly in the cytosol. This binding translocates the MR to the nucleus, where it acts as a ligand-induced transcription factor. However, it has been proposed that activated MR can elicit additional non-classical effects, which do not require transcription or translation of genes and involved the production of ROS (Caldiz et al., 2011), leading to the activation of ions membrane transporters (Ebata et al., 1999; Mihailidou et al., 2004; Chai et al., 2005; Grossmann and Gekle, 2009; Caldiz et al., 2011; De Giusti et al., 2011a). The mechanisms conveying theses rapid and non-genomics effects consist in several signaling cascades of kinases, as protein kinase C (PKC) (Ebata et al., 1999; Mihailidou et al., 2004) and ERK ½ (Caldiz et al., 2011), and also include a crosstalk with Ang II (Lemarie et al., 2008; Rautureau et al., 2011) and the transactivation of EGFR (Grossmann et al., 2010; De Giusti et al., 2011a). Moreover, it has been proposed the presence of a crosstalk between both, genomic and non-genomic pathways of aldosterone (Grossmann and Gekle, 2009). On the other hand, it was recently demonstrated that certain non-genomic effects of aldosterone in vascular smooth muscle were due to simultaneous activation of MR and a surface membrane G protein-coupled receptor, the GPR30 (Gros et al., 2011, 2013). In agreement, growing evidence is appearing which demonstrate that GPR30 could be another aldosterone receptor involved in the rapid effects of the hormone in the cardiovascular system (Gros et al., 2011; Meyer et al., 2011).

## Regulation of NBC by RAAS

It was demonstrated that Ang II stimulates total NBC activity, both in rat (Baetz et al., 2002) and cat (De Giusti et al., 2009) ventricular myocytes in a ROS- (De Giusti et al., 2009)and ERK ½-dependent manner (Baetz et al., 2002; De Giusti et al., 2009). Moreover, the phenomenon known as “ROS-induced-ROS-release,” where a small amount of ROS derived from NADPH oxidase (NOX) stimulates mitochondria to produce and release a burst of ROS (Zorov et al., 2000, 2006; Kimura et al., 2005), was also involved in Ang II-induced NBC stimulation (De Giusti et al., 2009).

Our group demonstrated for the first time a differential effect of Ang II on NBC isoforms through parallel pathways(De Giusti et al., 2010). Ang II after binding to AT-1 receptor inhibits NBCe1 in a p38 kinase-dependent, whereas activates NBCn1 via an ERK ½ and ROS-dependent mechanism (Figure 1). Furthermore, Ang II generated a higher increase in *J*_*H*_ in the presence of p38 kinase inhibitor than in its absence, leading us to the speculation that Ang II-induced stimulation of NBCn1 overrules the inhibition of NBCe1 (Aiello and De Giusti, 2013). Interestingly, Ang II abrogated the anionic current generated by the NBCe1 (De Giusti et al., 2010). It is well-known that Ang II induces CAP prolongation which leads to arrhythmic effects (Fischer et al., 2007). Thus, it is possible to speculate that this inhibition of NBCe1 by Ang II might be involved, at least in part, in the CAP prolongation and arrhythmias induced by this hormone (Figure 1).

It has been reported that aldosterone stimulates NHE-1 via MR in a non-genomic manner and via a ROS-dependent pathway (Caldiz et al., 2011; De Giusti et al., 2011a). As NBC activity was shown to be ROS (De Giusti et al., 2009) and ERK ½ kinase-dependent (Baetz et al., 2002), it might be possible to speculate that aldosterone can regulate NBC. Interestingly, Gros et al. have demonstrated that aldosterone mediates its rapid effects in vascular endothelial cells through GPR30 activation (Gros et al., 2013). Moreover, it was reported that GPR30 is expressed in the heart (Bopassa et al., 2010) and that the stimulation of this receptor by estradiol (Patel et al., 2010) or G1, a specific GPR30 agonist, (Deschamps and Murphy, 2009; Bopassa et al., 2010) mediates protection during ischemia-reperfusion injury (Deschamps et al., 2010). Whether the potential effect of aldosterone on cardiac NBC could be mediated through MR or GPR30 activation represents an interesting issue that deserves future investigation.

## Involvement of NBC in RAAS induced cardiac diseases

Aberrant H^+^-induced Na^+^ and Ca^2+^ influxes has been proposed to participate in maladaptive cardiac hypertrophy (Yamamoto et al., 2007; Baartscheer and Van Borren, 2008; Cingolani et al., 2008) and arrhythmogenesis (Baartscheer et al., 2008). In the last years, NBC disturbances were described during cardiac hypertrophy in close relationship with RAAS activation (Yamamoto et al., 2007; Orlowski et al., 2013). Moreover, there is enough information about the involvement of Na^+^ (Gonano et al., 2011) and Ca^2+^ signaling disorders (Venetucci et al., 2008) and Ang II stimulation (Zhao et al., 2011) in the genesis of arrhythmias. However, until present and besides NBC activity influence on [Na^+^]_*i*_ and [Ca^2+^]_*i*_, nothing is known about the direct involvement of NBC in arrhythmogenesis.

## Is NBC involved in cardiac hypertrophy?

Yamamoto et al have demonstrated that NBCe1 and NBCn1 were over-expressed in ventricular myocytes isolated from hypertrophied rat hearts subjected to non-ischemic pressure overload and that these changes were prevented by the AT-1 blocker Losartan (Yamamoto et al., 2007). However, a clear up-regulation of NBCe1 activity could not be demonstrated (Yamamoto et al., 2007). In addition, it has been reported that, although NBCe1 was also over-expressed in hypertrophied hearts of SHR rats, its activity was impaired (Orlowski et al., 2013). It was proposed that Ang II-induced NBCe1 internalization might explain the discordance between protein expression and transport activity (Orlowski et al., 2013) (Figure 1). Interestingly, in the same research it was suggested that NBCn1 activity was increased in order to maintain pH_*i*_. However, based in the different stoichiometry of both isoforms, this remodeling might lead to higher Na^+^ influx and in consequence greater intracellular calcium overload. In agreement with the hypothesis that NBCe1 could be internalized during cardiac hypertrophy, it has been described that Ang II promotes NBCe1 internalization in Xenopus oocytes transfected with this NBC isoform (Perry et al., 2007). Consistently, the differential membrane expression and function of NBC isoforms observed in the hearts of SHR rats were reversed by treatment of these hypertensive rats with losartan (Orlowski et al., 2013).

It is well-known that increased [Ca^2+^]_*i*_ activates hypertrophic pathways, such as the one of calcineurin (Ennis et al., 2007; Guo et al., 2011). Ca^2+^ regulation is closely linked to [Na^+^]_*i*_ because one of the routes for Ca^2+^ influx into the myocytes is via NCXr. During acidosis there is an intimate link between Na^+^ and Ca^2+^, mediated through a functional coupling among the activities of the alkalinizing transporters and NCX (Garciarena et al., 2013a).

In animal models of hypertrophy, as well as in human heart failure, it has been demonstrated an increase in [Na^+^]_*i*_ and [Ca^2+^]_*i*_ (Gray et al., 2001; Despa et al., 2002; Verdonck et al., 2003). Furthermore, it was shown that chronic NHE-1 inhibition, which attenuates [Na^+^]_*i*_ overload, restrains Ca^2+^ activated pro-hypertrophic intracellular pathways and reverses myocardial remodeling, leading to prevention or reversion of cardiac hypertrophy (Kusumoto et al., 2001; Camilion De Hurtado et al., 2002; Engelhardt et al., 2002; Ennis et al., 2003). Furthermore, it was demonstrated that the over-expression of NHE-1 alone is enough to induce cardiac hypertrophy in a murine model (Nakamura et al., 2008). Interestingly, these animals presented a higher NHE-1 expression, not only at intercalated discs, where it is normally expressed, but also all along the transverse tubules (Nakamura et al., 2008). Inappropriate trafficking of NBC may also be exacerbated by the increasing loss of t-tubules from ventricular cells in the progression to heart failure (Wei et al., 2010). In this regard, the normal selective spatial distribution of the alkalinizing transporters is crucial for the ion homeostasis, suggesting that the impairment of this distribution might be detrimental for myocyte function (Figure 2) (Garciarena et al., 2013a).

NBC is responsible for 30% of Na^+^ influx into the myocyte at pH_*i*_ 6.8 (Vaughan-Jones et al., 2006), so it may be also important to the development of cardiac hypertrophy. In this regard and as it was commented above, it has been shown that NBCn1 function is up-regulated in cardiac hypertrophy (Yamamoto et al., 2007), while NBCe1 transport seems to be impaired in the hypertrophied heart of SHR rats (Orlowski et al., 2013). Taking into account that NBCe1 has Na^+^-sparing activity, it is feasible to anticipate that this remodeling of NBC isoforms in hypertrophied hearts would lead to more [Na^+^]_*i*_ and [Ca^2+^]_*i*_ overload. Moreover, the distribution of NBCe1 along the transverse tubules clustered with the NCX and the sarcoplasmic reticulum (SR), the most important pool of Ca^2+^, proposes a relevant relationship between this NBC isoform and calcium handling. Furthermore, it was reported that luminal SR [Ca^2+^] is Na^+^_*i*_ sensitive, suggesting that the tubular NBC activity may allow to create a local control of pH_*i*_ while minimizing the effects of local Na^+^-loading on Ca^2+^ signaling (Garciarena et al., 2013a).

Another important aspect that should be taken into account is the possibility that the NBCe1 downregulation observed in SHR hypertrophic hearts may lead, not only to membrane overexpression of NBCn1, but also to an enhanced NHE-1 activity. Since a hyperactivity of NHE-1 in myocardium of SHR has been previously demonstrated (Perez et al., 1995), it might be interesting to elucidate if both phenomena are related and furthermore, if NBCe1 downregulation is the cause of NHE-1 hyperactivity in this strain.

## Is NBC involved in early and delayed after depolarizations (EADs and DADs)?

It has been shown that either the inhibition of the Na^+^/K^+^ ATPase (Sedej et al., 2010; Gonano et al., 2011) or the NHE-1 stimulation (Baartscheer et al., 2008) generates [Na^+^]_*i*_ overload which leads to cardiac arrhythmias due to the reduction of Ca^2+^ extrusion and/or the increase of Ca^2+^ influx through the NCX. The increase in [Ca^2+^]_*i*_ enhances the SR calcium load, leading to spontaneous diastolic calcium release. Then, the increase in cytosolic Ca^2+^ (waves) activates an inward (depolarizing) current (I_*ti*_), mediated by the forward mode of NCX (NCXf) (Bers et al., 2002; Rizzi et al., 2008). I_*ti*_ is responsible for the generation of the delayed after depolarizations (DADs) which, when are sufficiently large to achieve the threshold, generate spontaneous CAP, leading to triggered activity (Liu et al., 2011).

As NBC activity promotes the increase in [Na^+^]_*i*_ (Vaughan-Jones et al., 2006), it is also possible to speculate that Ang II and ROS-induced NBCn1 stimulation (De Giusti et al., 2009, 2010) might be implicated in DADs generation (Figure 1). According to this, it was demonstrated that Ang II induces DADs in a ROS-dependent manner (Wiederkehr et al., 1999).

Classically, Ang II is known to modulate the properties of ion channels involved in the cardiac action potential (CAP) configuration (Vila Petroff et al., 2000; Salas et al., 2001; Domenighetti et al., 2007; Rivard et al., 2008). It has been reported that Ang II prolongs CAP both, inhibiting repolarizing currents as I_K1_, I_Kr_, and I_to_ (Zhou et al., 2006; Domenighetti et al., 2007; Rivard et al., 2008; Wang et al., 2008) and stimulating depolarizing currents as I_CaL_ (Aiello and Cingolani, 2001; Ichiyanagi et al., 2002). Moreover, it has been recently demonstrated that Ang II abrogated the NBCe1-induced CAP shortening, likely due to the inhibition of the repolarizing current generated by the transporter (De Giusti et al., 2010). In this regard, it has been shown that CAP prolongation enhances the occurrence of early after depolarizations (EADs), due to the recovery from the inactivation and the reactivation of voltage-dependent L-type Ca^2+^ channels (Nuss et al., 1999; Wiederkehr et al., 1999; Xie et al., 2009) and the impairment of sodium current (Wiederkehr et al., 1999; Xie et al., 2009). In concordance, Ang II was shown to increase the occurrence of EADs in a ROS and CaMKII-dependent manner (Zhao et al., 2011), suggesting the possibility that Ang II-induced NBCe1 inhibition might participate in the generation of EADs.

In summary, it might be also possible that Ang II-induced NBCe1 inhibition and NBCn1 stimulation participate in the generation of EAD, secondary to CAP prolongation and [Na^+^]_*i*_ overload, respectively (Figure 1).

It is known that a close relationship exist between CAP prolongation and hypertrophy, but it is unclear which is cause and which the consequence. On the one hand, the CAP prolongation is consistently observed in several experimental models of cardiac hypertrophy and failure (Carmeliet, 2006). It is also known that this can lead to QT prolongation, which is the basis of arrhytmogenic events (Fischer et al., 2007; Baartscheer et al., 2008). On the other hand, Lebeche *et al* have reported that CAP prolongation promotes an increase in [Ca^2+^]_*i*_, which activates a hypertrophic signaling pathway that might be a cause and not a consequence of cardiac hypertrophy (Lebeche et al., 2006).

## Final conclusion

The purpose of this review is to focus the attention on the cardiac NBC and its regulation by RAAS, especially considering the implications of this modulation in cardiac diseases. Classically, NBC is known as an alkalinizing mechanism. However, it is important to keep in mind that this is not its only function but it also controls [Na^+^]_*i*_, and indirectly [Ca^2+^]_*i*_ through the NCX activity and SR behavior. Moreover, NBCe1 modulates the shape and duration of the CAP, adding to this isoform the important role of contributing to cellular electrophysiology. As RAAS exerts a central role in cardiac pathophysiology, it can be considered of significant relevance the fact that both hormones, Ang II and possibly aldosterone, regulate NBC activity. Moreover, Ang II seems to regulate the trafficking of NBC besides the influence in the transporter activity (Perry et al., 2007; Orlowski et al., 2013). Moreover, Ang II exerting an opposite effect on NBC isoforms, might be doubly detrimental, leading to Na^+^ and Ca^2+^ overload and CAP prolongation, which could be relevant, at least in part, to explain the hypertrophic and arrthymogenic effects of the hormone. Until present, it is not known if aldosterone can induce NBC stimulation via the activation of the electrogenic, the electroneutral or both isoforms of NBC. This issue might be kept in consideration because of the different role of each isoform in myocyte physiology.

The knowledge of the central role that sodium and calcium concentrations play and the close relationship between them and H^+^ movements with NBC expression and distribution, forces us to keep the attention in NBC and cardiac diseases. Furthermore, the fact that RAAS exerts direct effects on NBC activity strengthens the conviction that NBC might be responsible, at least in part, for the development and maintenance of cardiac diseases.

### Conflict of interest statement

The authors declare that the research was conducted in the absence of any commercial or financial relationships that could be construed as a potential conflict of interest.
